# Understanding what matters to young people with life-limiting and life-threatening conditions: a qualitative study to inform the development of a young person end-of-life capability measure

**DOI:** 10.1016/j.socscimed.2025.118335

**Published:** 2025-07-17

**Authors:** Isabella Floredin, Samantha Husbands, Susan Neilson, Paul Mark Mitchell, Joanna Coast

**Affiliations:** a Health Economics and Health Policy (HEHP@Bristol), Population Health Sciences, Bristol Medical School, University of Bristol, 1-5 Whiteladies Road, Bristol, BS8 1NU, UK; b Institute of Clinical Sciences, University of Birmingham, Edgbaston, Birmingham, B15 2TT, UK

**Keywords:** Capability measures, Conversion factors, End-of-life care, Young people, Life-limiting conditions, Life-threatening conditions, Qualitative methods

## Abstract

End-of-life care encompasses a range of services aimed at delivering holistic interventions. Yet, economic evaluations of such services have tended to be focused on health-related outcomes, which may be too narrow to adequately capture outcomes at the end of life. Capability measures aim to capture wider outcomes focusing on what people can do and be in their lives. Capability measures are not yet available for children and young people at the end of life. This study aimed to identify important capabilities for young people aged 14–25 years across the end-of-life trajectory, to inform attribute development for a capability measure for use in economic evaluation of interventions at the end of life. Twenty-one in-depth interviews were undertaken with young people aged 14–25 years with a life-limiting or life-threatening condition (n = 6), parents or guardians (n = 6), and bereaved family members (n = 9) in England and Wales between May 2021–September 2022. Interviews focused on what is important to the young people now and in the future. Constant comparative analysis methods were used to explore capabilities and factors influencing capability achievement (conversion factors). Seven capabilities were generated: Experience and enjoy; Independence; Freedom from physical suffering; Freedom from emotional suffering; Formal care and support; Control; Identity. Five conversion factors were generated: Access to care and support; Continuity and consistency in care and support; Communication with services; Coordination between services; and COVID-19. Findings can inform attribute development for a young person capability measure for use in economic evaluation capturing the broader outcomes of end-of-life care.

## 1 Introduction

End-of-life care (EoLC) aims to improve quality of life in the last months or year(s) of life through prevention or relief of suffering, whether physical, psycho-social or spiritual (World Health Organisation, 2020). EoLC provides a holistic approach with complex care interventions provided by a range of services and professionals (Coad et al., 2015; Stone, 2001). The importance of holistic outcome measures in EoLC to adequately address the multifaceted needs of individuals at the end of life (EoL) has been highlighted (de Wolf-Linder et al., 2019).

The assessment of outcomes in the evaluation of health and care interventions is, however, traditionally health focused (Rudmik and Drummond, 2013) and, in a number of countries including the UK, Canada, Australia, Thailand and the Netherlands (Kharroubi and Elbarazi, 2023), often relies on health-related quality of life (HRQoL) measures (Coombes et al., 2016; Morris et al., 2007). While these measures are widely used in economic evaluation to compare outcomes with costs across different interventions for the purpose of effective resource allocation in health care (Morris et al., 2007) they may be too narrow to adequately capture those EoLC outcomes that extend beyond health, such as preparation for death and spiritual needs (Stone, 2001). This critique regarding the narrow focus of HRQoL measures has also been made in other contexts, including social care (Hall and Kinghorn, 2022) and children with disabilities (Davis et al., 2018). The narrowness of measures means that, the effectiveness and cost-effectiveness of EoLC interventions may not be fairly assessed to inform decisions about care and resource allocation (Coast et al., 2016; Kinghorn and Coast, 2019; Normand, 2009).

Capability measures aim to capture wider quality of life outcomes focusing on people’s capabilities: the things people are able to be and do in life and which are of value to them (Nussbaum and Sen, 1993). Unique to the ‘capability’ concept, is the freedom a person has to achieve the things that are important to them, whether or not they choose to do so (Nussbaum and Sen, 1993). One aspect of the capability approach is to determine the attributes that are important to people and should be evaluated, and research has focused on this aspect. Another important element of the capability approach, however, is consideration of conversion factors. These conversion factors influence the processes by which a person is able to convert resources into achieved capabilities (Robeyns and Byskov, 2011; Sen, 1992). They may hinder or enhance a person’s capabilities and can be personal (related to personal characteristics), social (such as social norms and policies) or environmental (such as provision of a public good) (Robeyns, 2017; Sen, 1999).

The application of the capability approach to inform the development of measures for use in economic evaluation of health and care interventions is gaining traction (Helter et al., 2020) with capability measures proposed for use in decision making in the UK and the Netherlands, particularly around social and long-term care (National Institute for Health and Care Excellence, 2023; Zorginstituut Nederland, 2016). Only one such measure to date has focused on EoLC, the ICECAP - Supportive care measure (ICECAP-SCM) for EoL settings (Sutton and Coast, 2014). While the ICECAP-SCM captures the wider outcomes of EoLC, it was developed for use with older adults and the measure may therefore not be appropriate for children and young people (CYP) at the EoL (Harding et al., 2019). CYP capability measures are currently in development for different CYP age groups (Husbands et al., 2024a) but EoL is a different life stage, and so different capabilities may be important to CYP at the EoL (Huang et al., 2010). None of these measures explicitly consider conversion factors.

Beyond physiological and developmental differences between adults and CYP, there are differences in the illness trajectories and types of life-limiting and life-threatening conditions (LLCs/LTCs) (Chelazzi et al., 2023). LLCs are conditions for which there is no hope for a cure and CYP will live a short life (Knapp et al., 2012). For LTCs treatment may be possible but it may fail (Knapp et al., 2012). CYP with LLCs/LTCs may receive palliative care (PC) and EoLC for a longer period of time than adults, and this may also involve a transition from child to adult services at ages 18 years+ (World Health Organisation, 2020). This transitional period for YP aged 14–25 years, is particularly challenging due to differences in the nature of care provision between children and adult PC and EoLC services (Chambers, 2015; Fraser et al., 2020).

While there are unique ethical and practical challenges in exploring what is important to CYP at the EoL (Harding et al., 2019) there is a need to develop appropriate measures for this population to be able to assess EoLC outcomes for CYP for use in economic evaluation. Given the particular challenges that YP experience during transition to adult services, the work reported here is focused on outcomes for YP aged 14–25 years at the EoL.

Qualitative research was undertaken in the UK and aimed to: a) identify important capabilities for YP with LLC/LTCs approaching the EoL to inform the conceptual attributes for a young person capability measure for use in economic evaluation of care at the EoL and b) identify the conversion factors associated with achieving these capabilities.

## 2 Methods

### 2.1 Study design

Qualitative methods were used to capture the views of YP with LLC/LTCs coming towards the EoL and their family members. Online, in-depth interviews explored aspects of life that are important to YP (important capabilities) at the EoL to inform the attributes of a young person EoL measure (J. Coast et al., 2012; Pickles et al., 2019). Compared with attribute development based on the views of the researcher or ‘experts’, the involvement of the relevant population can improve the face and content validity of the final measure (Stevens and Palfreyman, 2012). Data collection and analysis were undertaken concurrently and analysed using constant comparison (Glaser and Strauss, 1968; Strauss and Corbin, 1990).

Study methods are reported in accordance with the consolidated criteria for reporting qualitative studies (COREQ) (Tong et al., 2007) (see Supplementary File 1).

The work reported in this paper was part of a wider study focusing on capability measurement across the life course for use in economic evaluation [details of reference anonymised].

### 2.2 Participants and recruitment

Initial recruitment focused on YP aged 14–25 years with a LLC/LTC or their parents/guardians (initial study inclusion criteria) to identify important capabilities at the EoL for YP. The Directory of LLCs/LTCs (Hain et al., 2013) was used alongside advice from the research team with expertise in paediatric palliative care (PPC) (S.N.) to identify potential participants with LLC/LTC. Parents/guardians were included as proxy participants where YP were not able to take part directly due to their condition and/or symptoms (YP therefore not present during interviews). The voices of YP who are at the EoL cannot be replaced with those of others. In their absence, however, the research team felt that parents/guardians would be the most appropriate group of proxy participants to complement the voices of these YP, and in particular the voices of YP at the very EoL. Parents may provide a more holistic experience of the young person’s life compared to other potential participant groups, such as health care professionals or teachers, whose views may focus on specific topics such as physical health (Coombes et al., 2023) or education.

During the recruitment and analysis stages, as better understanding was gained about the YP’s experiences and conditions, participant inclusion criteria shifted to also include bereaved family members (parents/guardians and siblings) of YP aged 14–25 years who had died from a LLC/LTC. This inclusion was intended to further facilitate acquisition of the experiences of YP at the very EoL and also helped to increase recruitment more generally, as recruitment had initially been slow.

A maximum variation approach to sampling (Patton, 2015) was intended to capture different perspectives of YP from a broad range of socio-economic backgrounds (determined using data on age, sex, ethnicity, Index of Multiple Deprivation (2019) (IMD) (Jones, 2019; Noble et al., 2019), conditions, care settings and illness trajectories (captured through the use of a pre-interview questionnaire). Recruitment strategies aimed to facilitate this maximum variation approach. Given the sensitive topic and the vulnerable participant group, however, the number of potential participants was anticipated to be small and therefore all those who came forward for interview, who fit the inclusion criteria, were included. Snowball sampling through existing participants was also used. To maximise recruitment, multiple avenues were used to recruit participants across the UK. For example, online and printed adverts were used, as well as a study newsletter circulated via staff members, by email and/or in-person, in child (four) and adult (one) hospices, charitable organisations (six) and through social media using Twitter posts, relevant support groups on Facebook and the Facebook ‘targeted ads feature’ (see Table 1 for numbers recruited through different routes).

Recruitment materials for YP and parent/guardian participants were developed separately and tailored to aid understanding. Feedback was sought on draft recruitment materials (adverts and information sheets) from two YP’s advisory groups, one with YP aged 16–18 years with LLC/LTC. The layout and language of the final materials were adjusted following advisory group feedback. Advisory groups were not used to aid recruitment.

During recruitment, potential participants were asked to read an information sheet stating the aim of the study and what taking part involved, prior to making their decision about whether to take part.

In line with other attribute development studies, recruitment ended when the conceptual attributes were deemed to be fully developed (J. Coast et al., 2012; Owen-Smith and Coast, 2017).

### 2.3 Data collection

Interviews were undertaken by (I.F.) during the COVID-19 pandemic (May 2021–September 2022). I.F. had previous experience working and undertaking research with vulnerable groups and received training on conducting interviewing on sensitive topics. Most interviews were undertaken online and video and audio-recorded. Some participants preferred a telephone interview and this was also facilitated. At the time of their interview, participants were located in either home or care settings. YP were given the option of parents being present, for example to assist with using technology to take part in the online interview and to interpret non-verbal communication.

At the start of the interview, it was stressed to participants that they could stop the interview and/or withdraw from the study at any time. Interviews started with warm up questions asking the participant to say a little bit about themselves. Warm-up questions aimed to: i. help participants to feel comfortable and ii. provide context (such as family background, hobbies, medical condition and symptoms, care currently received) before moving onto the main interview questions to explore aspects of life which are important to YP at the EoL. Three separate topic guides were used (Supplementary file 2). The topic guide for YP asked questions about their health condition and aspects of life that were important to them and why. The topic guides for family members asked questions about the young person’s condition and what aspects of life they thought were important to the young person and why. All topic guides were updated iteratively throughout the data collection period to enable important topics to be covered fully during interviews.

Reflective notes following interviews helped explore what matters to YP at the very EoL as early YP participants did not talk about this in detail. The researcher adjusted their approach, starting with general questions about participants and their condition which made it easier to ask more tailored questions about key EoL topics like prognosis and condition progression. Transcripts were not returned to participants and were only seen by the research team.

### 2.4 Data analysis

Interviews were transcribed verbatim and uploaded into a qualitative data analysis software (NVivo12). Analysis was undertaken primarily by I.F. with support from research team members (J.C., S.H., S. N., P.M.) with disciplinary backgrounds in the capability approach (J.C., S.H., P.M.), economics (J.C., S.H., P.M.), qualitative research (J.C., S.H., S.N.) and PPC (S.N.). Analysis was undertaken in batches of two to three transcripts depending on the dates that interviews took place and to allow for topic guides to be updated iteratively. Early transcripts were coded on a line-by-line basis (open coding) (Strauss and Corbin, 1990) by I.F., S.H., S.N. to identify emerging themes in terms of what is important to YP at the EoL. Codes were developed in a hierarchical manner, comparing data for similarities and differences (axial coding) (Strauss and Corbin, 1990). Three separate coding schedules were then developed for each group of participants (1. YP; 2. Parents/Guardians; 3. Bereaved family members) which were updated iteratively. Independent open coding by three members of the research team facilitated comparison of the coding data identified by each researcher to develop the initial coding schedules alongside spider diagrams to map initial themes and sub-themes included in the schedules. Eight descriptive accounts were developed to facilitate theme comparison between each of the three groups. Descriptive accounts were brought together in a final analytic account repeating comparison of key themes between participant groups (Jackson, 2017). Constant comparison of data (Glaser and Strauss, 1968) was conducted until no new themes arose on what appears to be important to YP at the EoL.

To ensure that the capability themes were focused on informing attributes for a future measure, themes specific to the context of COVID-19 were coded and presented as a distinct theme. This approach supported the analysis in ensuring that the capability themes remained as unaffected as possible by the contextual influence of COVID-19 while still acknowledging its significant impact on participants’ experiences.

### 2.5 Ethical considerations

The study obtained ethical approval from the University of Bristol, Faculty of Health Science Research Ethics Committee (FREC) (FREC reference number: 111822).

For ethical reasons, bereaved family member participants who were within 6 months of bereavement were not recruited in this study to avoid potential early intense grief experienced (Canaway et al., 2016; Hynson et al., 2006).

Prior to the interview, written informed consent was obtained from participants aged 16 years and over. Written informed consent was obtained from parents/guardians of participants aged under 16 years, alongside assent from the young person. This ensured the YP participants were happy to take part and had the opportunity to ask any questions (NHS Health Research Authority, 2021).

To manage potential distress, a protocol was developed with clear plan for the interviewer to follow if a participant became distressed. As part of the study’s debrief, participants were provided with information and contact details of free support services. To manage the emotional impact of the work on the researcher, reflections were recorded after each interview and discussed at regular debrief meetings with members of the research team (J.C., S.H., S.N.), including researchers who have experience of sensitive interviewing including at the EoL.

Care was taken to anonymise identifiable participant information from transcripts. This included their health condition, symptoms and treatment, due to some of the conditions being rare and/or specialised treatment being received. Medical conditions were replaced on transcripts with the relevant diagnostic group category, per the categories of LLCs/LTCs outlined by Fraser and colleagues. (Fraser et al., 2020).

### 2.6 Presenting the findings

Illustrative quotes are provided to support each theme. Ellipses (…) are used within quotes to indicate missing text. Context is clarified using square brackets […] within quotes. Quotes from YP are indicated by ‘YP’, quotes from parents/guardians by ‘PG’ and quotes from bereaved family members by ‘BFM’.

## 3 Findings

Forty-five people expressed initial interest in participating, from which twenty-one interviews were undertaken with six YP, six parents and nine bereaved family members (parents and siblings). Participant characteristics are in Table 1. Interviews captured the experiences of YP from diverse backgrounds, including those from the most deprived areas (n =11), with a wide range of conditions, illness trajectories and types of care received.

The most common reasons for individuals not being included in the study after expressing initial interest were that they did not fit the age criteria (n = 6), their condition was not a LLC/LTC (n = 7) and the bereavement period was less than six months (n = 2). Some did not respond to follow-up contact (n = 8).

Twelve interviews were conducted online and nine by telephone. Parents were present in two interviews with YP. Four participants took breaks during their interview, due to either feeling tired or finding the topic of discussion difficult, but were happy to finish the interview. Interviews with YP lasted between 27 and 47 (mean 37) minutes; those with family members lasted between 11 and 68 (mean 44) minutes.

### 3.1 Distinction between capability and conversion factor themes

**Fig. 1:**
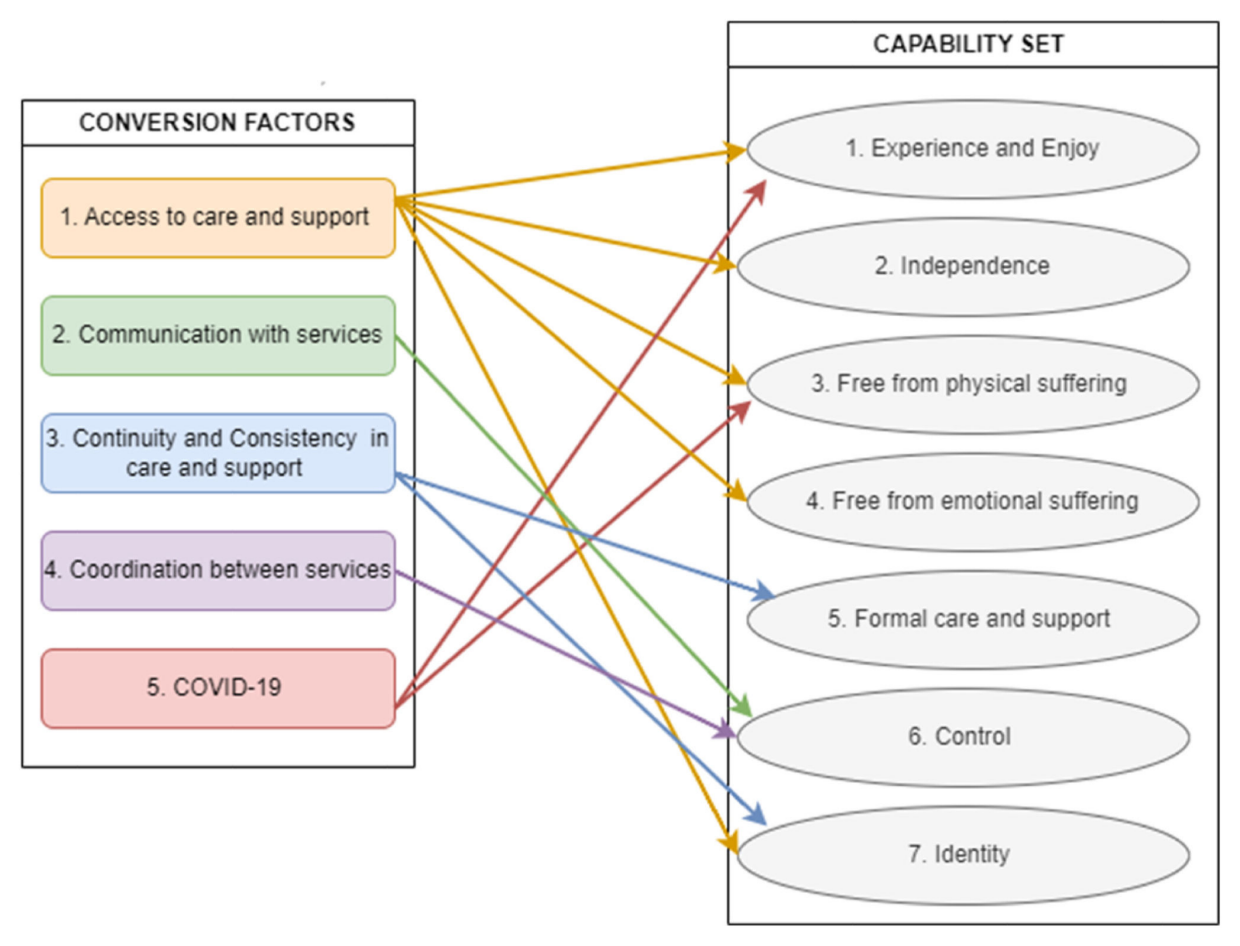
Capability and conversion factor themes.

Comparison of themes within and across the three participant groups enabled, through the analytic process, a distinction between themes relating to the capabilities of intrinsic value to participants (n = 7) (Sen, 1992), and those relating to conversion factors that hinder or facilitate the ability to achieve these capabilities (n = 5) (Robeyns and Byskov, 2011). Where themes held roles as both capabilities and conversion factors (for example, being free from physical suffering is of intrinsic value but also of value to be free from emotional suffering) they were categorised as a capability. An illustrative figure (Fig. 1) summarises how conversion factors relate to capabilities.

### 3.2 Capability themes

Seven capabilities for YP aged 14–25 years with LLC/LTCs were developed and are presented below.

#### 3.2.1 Experience and enjoy

A recurrent theme identified across all three groups of participants was the importance of being able to have as many experiences and as much enjoyment in life as possible. Participants described YP wanting to actively participate in and seek out meaningful life experiences. Experiences included activities that YP enjoyed doing with others, such as sports, or on their own, such as listening to music. Living with uncertainty was highlighted in many accounts, with participants suggesting that it was important to have as many opportunities as possible to enjoy life in the present and to be able live ‘a normal life’ as much as possible. For most, this meant a life beyond the medical diagnosis and associated care, which was particularly important when spending long periods of time in a clinical or care setting.

***YP02:*** *“… The future is unpredictable, you don’t know what’s gonna happen, you could be fine one minute and ill the next, and I feel like life is very short not to do it. …”*

***PG05:*** *“… we park in the van and sit with her in the van. And if it was warm she’d be on the side of the rugby pitch. […] We try to give her a normal life …”*

***BFM09:*** *“… to enjoy life around the medical (…) as much enjoyment as we could possibly give her. Opportunities, and outings, and special moments (…) She wanted to get out of the hospital (…) to see new places and do things.”*

#### 3.2.2 Independence

It was suggested across all participant groups that being independent was important. Independence was about not having to rely on other people to do daily living activities, such as washing and getting to places, and having privacy. YP wanted independence but often struggled with needing constant support. Their expressed frustration highlighted their desire to be independent.

***YP08***: “*… I’ve been completely reliant on my parents because I can’t do it [get to places by] myself …”*

***YP12:*** *“… I’ve got someone with me all the time, coming around, having to carry my bag, getting me around the doors, if I need to go to the toilet and stuff …”*

Parents and bereaved family members appeared to recognise this desire from YP for independence and suggested that independence was particularly important to YP so that they can enjoy life like other YP at their age do.

***BFM13:*** *“… she couldn’t do anything (…) it was difficult for her to make herself food. Live an independent life, like a normal 15-year-old would ….”*

As YP neared the very EoL, the loss of independence became even more apparent with YP wanting to maintain their independence despite increasing limitations.

***BFM18:*** “… *she really wanted to be able to have that independence. Especially when she became very unwell in the months before her death, she wasn’t able to do much at all …”*

#### 3.2.3 Freedom from physical suffering

Physical symptoms varied significantly across YP but included: pain, exhaustion, breathing difficulties, visual, hearing and/or mobility impairment. Managing physical symptoms was important to avoid further suffering and distress, including intensive care. As YP approached the very EoL (as indicated particularly in the accounts of bereaved parents), physical symptoms appeared to become more severe and complex and required more careful management to avoid suffering.

***YP01***: *“… go see doctor regularly … it’s keeping me safe and alive …”*

***PG11:*** *“… we don’t get to crisis point. We manage her [chemical that body produces] (…) and she doesn’t need acute, critical care because of how she’s looked after …”*

***BFM06:*** *“… in hospital she had a care plan, because her skin was really puffy where she was holding on to fluid, and it was really sore. And she had a particular care plan to do with moving her in the bed and looking after her skin …”*

While in bereaved parent accounts, details of physical symptoms were more prominent, YP and parents talked more about how the symptoms impacted the young person’s daily life, independence, and ability to enjoy life. As a result, physical symptoms were seen as having a significant impact on emotional and mental wellbeing.

***YP02:*** *“… I worry about getting ill and getting a chest infection because I am prone to them and it does make me nervous (…) I have not been able to eat some of types of food, that made me really sad …”*

#### 3.2.4 Freedom from emotional suffering

All YP and most parents and family members talked about emotional wellbeing and the focus appeared to be around coping mechanisms for alleviating emotional burden, especially near the very EoL and when YP had to be in hospital, times more likely to be discussed by bereaved family members. Having a serious illness, often with an uncertain trajectory, had a significant impact on the YP’s emotional wellbeing. YP had different ways to cope with distress and difficult emotions including using distraction and sharing worries with others to offload. Some YP and bereaved family members suggested that helping others who are also struggling helped the YP to feel useful.

***YP03:*** *“… [listening to music] helps me relax (…) it makes me forget about certain things (…) because things can get overwhelming sometimes, so I just like to let my mind loose and do what I like to do …”*

***YP12:*** *“… I’d write the song completely how I’m feeling (…) maybe hopefully it works with others and people maybe feel the same …”*

Towards the final stages of life, emotional suffering often intensified and bereaved family members described this in detail, particularly in relation to long hospital stays.

***BFM09:*** *“She was really down and just sick of being stuck in there … [In hospital] they brought these virtual reality goggles to her, and she was able to watch, an underwater world, so basically making them feel like they’re not in hospital …”*

#### 3.2.5 Formal care and support

The YP’s experiences of formal care and support from health and social care services and in educational settings was talked about in detail. In these settings, it was important to YP to feel supported and cared for by trusted care providers.

***YP08:*** *“… That makes a difference. I feel like they [medical team] remember me when I come in (…) I feel familiar with them all (…) I feel very attached to all of my medical professionals. But that helps because obviously you trust them with a lot of stuff …”*

***BFM09:*** *“… People [in hospital] that were willing to give her the time and sit down and spend a little bit of time with her in talking about things and getting to know her. …”*

#### 3.2.6 Control

Another strong theme around care and support was around the YP feeling in control over their health and care. Important aspects of feeling in control for YP were around being able to have a say, be listened to and being able to make their own choices regarding their health and care.

***YP01:*** *“… being able to do my own choices …”*

***YP03:*** *“… as much as anyone else thinks what I need is best, then sometimes it’s actually helpful to ask the person who needs the help (…) I’d like it if there was more of me having a say in things (…) when I turn 18 I’ll be able to have a lot more say in what happens …”*

***BFM06:*** *“… She wasn’t happy about being in hospital (…) She had had enough of it. But she knew she had to be there. So they just had to do things her way …”*

As YP neared the very EoL, bereaved family members reported that feeling listened to and having their concerns taken seriously became even more important to the YP, yet this was not always their experience.

***BFM18:*** *“… she was very unwell towards the end (…) she felt that she wasn’t really being listened to, that she tried speaking to them [doctors] (…) but they reassured her there’s nothing seriously wrong …”*

#### 3.2.7 Identity

Identity was another important theme. Some YP were concerned about how their disability and health challenges might shape others’ perceptions of them, particularly the fear of being pitied or judged because of their disability rather than being seen and valued for who they were as a whole person.

***YP12:*** *“… it’s always in the back of my mind if actually people take me seriously or they’re just pitying me …”*

***BFM13:*** *“… she didn’t want to be invisible. Just because she was unwell didn’t mean she wasn’t relevant. And she had a value and something to say. And I think, because she had a walking stick as well, she was very worried she’d be judged for being young …”*

Other YP suggested that disability was part of their identity, and they wanted to spread awareness and challenge how people with disabilities are viewed by society. For example, one young person explained how being a disability advocate allowed them to challenge stereotypes about people with disabilities.

***YP02***: “*… to spread awareness of disability, so trying things like being a disability advocate on [social media account] (…) Because I feel like disability and disabled people do get pushed aside (…) just proving that disabled people are not all sad and gloomy … that is what people view us as in society …”*

In both cases, what was important to YP was to be valued and included for who they are as a person beyond the condition and/or disability.

The loss of identity and the need for recognition of the young person beyond their condition appeared to be particularly prominent near the very EoL where bereaved participants described challenges faced by YP in maintaining a sense of self when they were physically very unwell.

***BFM06:*** *“… [Near the end of her life] they [healthcare professionals] just saw this very sick child, just lying there, not particularly responsive (…) She lost who she was (…)there needed to be a lot more understanding of the person that she is, not the person that they could see that is just lying there very, very poorly …”*

### 3.3 Conversion factor themes

Five conversion factor themes, i.e. factors which hinder or enhance the young person’s ability to achieve important capabilities, were generated and are presented below.

#### 3.3.1 Access to care and support

YP often missed out on opportunities for enjoyable experiences due to not being able to access the support needed (e.g. financial) to attend activities. Parents, often having to ‘fight’ to access necessary support for their child, expressed frustration with how lack of access to support limited opportunities for YP to enjoy life to the full.

***PG01:*** *“… he wants to learn music. He can’t just go into a music college and enrol there because he has to have help at the college (…) and it’s kind of, people not able to give the money and services that he needs, which is quite disgraceful …”*

Parents were also frustrated with how lack of access to necessary health care impacted the young person’s physical health. As with access to practical support, parents reported having to ‘fight’ to access necessary care to avoid further suffering for their child.

***PG04:*** *“… Trying to access equipment and vital things for your child. You’re always met with an assessment to basically see if your child has the monetary worth to get it, or not. It’s always a fight. It’s a fight for everything (…) He’s never been without care because I’m always there…”*

Many parents and bereaved family members suggested that it was important to be able to access social care, such as house adaptations, so that YP could maintain independence.

***PG03:*** *“… House adaptions (…) They [social care services] are trying to do things which are not suitable, and (…) [son] had to move out of our house and live with [his grandparents] in their living room because the house wasn’t adapted to go to the toilet …”*

The lack of access to age-appropriate services for YP transitioning to adult services was suggested by parents and bereaved family members to have a negative impact on the young person’s identity and sense of belonging. This was because the YP felt too old to be in child services but too young to be in adult services.

***BFM07***: *“… She was in an adult ward. And, with it being a heart issue they were nearly all elderly. (…) So that made her feel even more of an odd bod …”*

***BFM13:*** *“… [In child services] It was difficult for her sometimes, because (…) she was 16, 17 and there were little 3, 4-year-olds and babies running around (…) And she was sort of sitting there with little Disney characters drawn on the wall. She felt a bit out of place …”*

Access to care and support from informal sources was also important. Being able to have emotional support from family and friends, and in particular from people they could trust, was valued by YP, and had a significant impact on their emotional wellbeing.

***YP02:*** *“… family are always there for you and they are a great support system that you can have (…) [they] support me when I am feeling very down, cheering me up (…) they are always there …”*

*YP12: “… I never really managed to get anyone [friends] and I couldn’t really trust anyone throughout my whole time because no one would speak to me (…) It’s about people spending time, dedicating time to you…”*

Close family was the main source of consistent informal support for many YP while, in many of the accounts, participants suggested that YP were let down by friends.

***BFM07***: *“… Her friends, really didn’t come through for her, and she felt quite let down by them. In the end all she wanted to see was family. And only a limited number of them. So she didn’t really find too much* support *outside of that group …”*

#### 3.3.2 Continuity and consistency in care and support

Parents and YP talked about the importance of having continuity and consistency in care and support, so that there was time to build trust with people involved in the young person’s care. Continuity and consistency were particularly important during transition to adult services when YP had to get used to a new team and the staff members who were new to the YP needed time to get to know them, their individual preferences and needs.

***PG04:*** *“… There just needs to be some continuity with staff (…) [In transition to adult services] we lost all his child team and we were introduced an adult team. So it was really difficult because some of his carers had been looking after him since he was nine months old (…) then all of a sudden they weren’t there anymore …”*

***YP08:*** *“… It was good to be out of hospital. I knew this coming though so I was prepared. I knew the community weren’t doing much. You’re just by yourself in this …”*

#### 3.3.3 Communication between the family and services

YP and parents suggested that good communication between the family, YP, and health and care services is important so that YP can feel in control. For most participants, good communication meant: being given adequate information about the condition, care and treatment options; information being communicated in lay language that YP and the family could understand; and having a chance to ask questions.

***PG01:*** *“… Some [doctors] talk big language that he doesn’t understand it so it’s (…) a barrier in the way he understands and that does confuse him (…) he will say that “I don’t understand them …” they “don’t talk my language” …”*

***YP12:*** *“… there are still so many issues with my health, I don’t have time when I speak to the doctors. I’ve got so many questions …”*

Some YP did not find being given detailed information regarding their health and care was always helpful and it was important that information was communicated at the right time, in the right amount and to the right person.

***BFM07:*** *“… you have got to make a decision, but sometimes (…) I would have appreciated being told things [by doctors] when I felt ready to. In fact, I think I did say, “Can you not tell me any more about that? Because I don’t know what to do with it. You are just telling me, and I can’t do anything about it. …”*

***BFM17:*** *“… if the nurses or the doctors (…) got all the stuff ready and then called him into the room and literally just (…) did whatever was needed, he would let them. (…) Whereas, if they’d try and talk to him and try to tell him what was happening, and he would just get really anxious …”*

#### 3.3.4 Coordination between services

The importance of good coordination between the different services was also highlighted predominantly in parent accounts, with parents suggesting that coordination was important so that YP could feel in control over their care. Parents felt that they often had to take a ‘care coordinator’ role to facilitate communication and coordination between the services involved in their child’s care. It appears that there was an increasing need for parents to take on this coordinating role during and following transition to adult services where they perceived fewer services as being available and an unrealistic expectation for YP in this situation to take on the role themselves.

***BFM09:*** *“… [to prepare for and following transition to adult services] a key doctor or a key person would be really helpful to them because, especially with my little girl, she may have been 18, but she wasn’t mentally 18. She wasn’t ready to coordinate all the stuff by herself ….I was having to coordinate everything …”*

***PG03:*** *“… you lose all your support from the age of sixteen where it just seems that there is this gap, between sixteen to eighteen, because children’s services don’t wanna know them because, they are going to be turning into adults, and adults don’t wanna touch them because they haven’t turned eighteen …”*

While it was important for YP to make decisions and feel in control over their care the reduced support and poor coordination evident in parental concerns about the transition to adult services influenced how much control the YP could realistically exert, with parental intervention often being needed.

#### 3.3.5 COVID-19

As these interviews were undertaken during the COVID-19 pandemic, many participants talked about two ways in which that context had influenced the YP’s capabilities. First there was a significant impact of the pandemic in limiting the young person’s opportunities to enjoy life.

***BFM06:*** *“… during COVID it was really hard for her (…) when she was in hospital she was fine as long as there were people around her. As long as there were visitors (…) I was there all the time, so she got bored of me. She wanted other people to be there …”*

***YP02:*** *“… before coronavirus and that, we used to be able to go out … we used to go to the cinema …”*

Second, during the first two years of COVID-19 (2020–2021), the ability to access necessary care and support worsened and the need to ‘fight’ for services reported by parents increased as medical appointments, especially in-person, became less regular.

***PG03:*** *“… Covid had a knock-on effect because all of [his] consultants stopped seeing him so his condition had deteriorated …”*

## 4 Discussion

To identify broader outcomes that are important to YP at the EoL interviews were undertaken with YP with LLCs/LTCs and family members. Seven capability themes were generated, which can be used to inform attribute development for a measure of capability wellbeing for YP at the EoL: Experience and enjoy; Independence; Freedom from physical suffering; Freedom from emotional suffering; Formal care and support; Control; Identity. Five conversion factor themes were generated which influenced the capabilities of these YP: Access to care and support; Continuity and consistency in care and support; Communication with services; Coordination between services; COVID-19. Whilst most conversion factors are likely to have enduring impact, the latter was specific to the timing of the work, during the COVID-19 pandemic.

The capability themes were broadly the same across the three groups of participants and the range of conditions captured in the study, including both malignant and non-malignant conditions. Despite the heterogeneity of LLCs/LTCs in CYP, what these YP appear to share is the unpredictable nature of LLCs/LTCs (Hynson et al., 2003) and the life uncertainty highlighted in the Experience and enjoy theme. This uncertainty may explain why what is of intrinsic value does not seem to vary across conditions and why no future-focused themes, such as future planning, emerged. This contrasts with future-focused themes in other studies on CYP with LLCs/LTCs (Coombes et al., 2022; Sodergren et al., 2018) and may reflect the EoL focus of this study. This is reassuring with regards to using these data to inform attributes for a YP EoL measure.

There were some differences regarding the importance of certain themes across the participant groups. Physical suffering was more prominent in bereaved parent accounts. It is possible that physical symptoms were more severe near the very EoL. This may reflect the heightened importance that family place on being able to manage symptoms at the very EoL stage or that the young people directly interviewed (or parents as proxy) had not yet reached a stage of experiencing more severe physical symptoms. Conversion factor themes were more prominent in parent accounts, as they often described ‘fighting’ for services for their child and coordinating communication between services, echoing past research (Fisher et al., 2023; Price et al., 2024). This focus likely reflects parents’ central role in managing these challenges, which YP described less frequently.

The capability themes of Physical and Emotional suffering, Independence and Identity share similarities with themes in previous qualitative studies undertaken with CYP with LLCs/LTCs and/or family members (Coombes et al., 2022; Mitchell et al., 2021). Themes also share similarities with condition-specific studies, for example around identity and disability (Gibson et al., 2014; Knox et al., 2017; Peat et al., 2023) and around control (Garland et al., 2020). Yet, in these existing studies there is no explicit distinction between what is of intrinsic value (capabilities) and the factors influencing capability achievement (conversion factors). This distinction is of particular importance to the development of a measure, as it is the capability themes, rather than the conversion factors, which will inform the conceptual attributes (Alkire, 2015).

Compared with dimensions of existing HRQoL child measures, such as PedsQL (Varni et al., 1999), CHU-9D (Stevens, 2010), EQ-5D-Y (Wille et al., 2010), C-POS (Coombes et al., 2024) and adult PC measures, such as POS-E (Dzingina et al., 2017), many aspects of life reported as important in the accounts are not captured in existing measures, such as Experience and enjoy, Control and Identity (See Supplementary File 3, Table 1). This is not surprising given that these measures have a health-related outcome focus. Important outcomes may therefore be excluded within evaluation if using existing measures in the EoL context for YP.

The attributes identified share similarities with both the dimensions of an existing adult EoL capability measure (Sutton and Coast, 2014) around themes of ‘independence’, ‘control’, ‘care and support’, and those of a child capability measure (ages 11–15 years) with similar themes of ‘enjoyment’, ‘experiencing’, ‘identity and choice’ (Husbands et al., 2024b) (See Supplementary File 3, Table 2).

Yet, there are some differences in the work presented in this paper. The theme of Experience and enjoy is not present in the adult EoL measure and the theme around Formal care and support is not present in the child measure. It is also of note that while the theme of attachment has been found in a child capability measure (‘love and friendship’) (Husbands et al., 2024b) and an adult EOL capability measure (‘love and affection’) (Sutton and Coast, 2014), attachment does not appear as a standalone attribute in the work presented in this paper. Yet, related aspects, such as connectedness and supportive relationships with family and friends, are present in the theme of Freedom from emotional suffering and the theme on Access to care and support, around informal support from family and friends. The disappointment expressed in many accounts around YP being able to maintain friendships and the family focus on, and fight to, access formal support may help explain the lesser focus given to aspects of attachment as an important factor in these YP’s lives. While this lesser focus on attachment could be a result of participants consciously accepting and adapting to the situation, it is possible that participants subconsciously adapted their views and values to fit the limited options they perceived were available to YP regarding attachment (adaptive preferences) (J. Coast et al., 2018). Nevertheless, these differences reiterate that important capabilities may differ across the life-course, with EoL in YP providing a situation in which two parts of the life-course are conflated.

A clear strength to the methodology used in this paper lies within the direct primary research undertaken with a hard-to-reach group on a sensitive topic. This was achieved despite the ethical challenges (Peake et al., 2022) in undertaking research with this population, albeit with recruitment that was relatively slow with a small number of potential participants. As a result, all initial respondents were included in the study. Despite the recruitment challenges, participants were from a range of backgrounds, including from the most overall deprived areas, and had a range of conditions. The use of multiple recruitment avenues was also an important strength as it allowed inclusion of participants who had access to a range of services.

Recruitment challenges were most evident in recruiting YP and in particular, YP at the very EoL. It was difficult to explore what was important at the very EoL with young person participants because of uncertainties about the timing of death, the feasibility of capturing interviews at this time and the desire of the YP at times to focus on aspects of living rather than dying. For practical and pragmatic reasons, the study adjusted the sampling strategy and used an overview of different perspectives with an overall aim of capturing what is important to include in a measure for YP at the EoL. The inclusion of these different perspectives resulted in a more rounded approach that could incorporate the perspectives of YP, both verbal and non-verbal, and across the EoL trajectory, including at the very EoL.

The work presented in the paper aims to inform the development of a capability measure for the assessment of broader outcomes of care for YP at the EoL. Further work is necessary to develop the measure. The capability themes generated can be taken forward to determine the final attributes that would form the measure dimensions. As with previous qualitative work to develop capability measures, further interviews with the relevant population should be undertaken to develop item wording and check that meaningfulness of the content and coverage of the attributes to the YP who will be completing the measure (Canaway et al., 2016; Sutton and Coast, 2014). Given the possibility of adaptive preferences related to the theme of attachment, assessing the content and coverage of attributes will also help determine whether attachment should be included as a standalone theme in the final measure. Furthermore, the psychometric properties of the final measure will need to be tested and valuation work undertaken before the measure can be used in economic evaluation.

Although the study aim was to develop capability attributes for a measure for economic evaluation, the conversion factors generated provide valuable information on what is important to capability achievement. In future work, it would be valuable to collect information about conversion factors alongside capability measures to explore their influence on the capabilities of different individuals. This information is likely to aid interpretation of the capability information and understanding of how different factors influence levels of capability.

Given the lack of appropriate measures for CYP at the EoL, future research is also necessary to explore outcomes for younger CYP groups at the EoL. It is possible that the different life stages and developmental changes in childhood may influence what aspects of life are valued across different ages. Furthermore, potential differences in cultural context and health and care systems in non-UK settings need to be considered.

Findings of this work could also inform outcomes for YP with disabilities and those with LLCs/LTCs beyond the EOL context. Davis et al. (2018) highlight the importance of using measures that focus on well-being rather than functioning or health limitations, consistent with a rights-based approach and the Convention on the Rights of Persons with a Disability (CRPD). This further highlights the need for measures that capture meaningful dimensions of life for these groups. Further research is needed to ensure all relevant dimensions for these groups are fully captured.

In conclusion, this study has made a novel contribution in the area of assessing outcomes of interventions at the EoL. There is paucity of measures that can be used to measure outcomes of care at the EoL for YP. The empirical work in this paper has addressed this gap within the literature by conducting primary qualitative work to inform measure development. This is the first qualitative study to capture capabilities that are of intrinsic value to YP with LLCs/LTCs and will help to move forward the area of economic evaluation for health and care interventions at the EoL.

## Supplementary Material

**Appendix A. Supplementary data**

Supplementary data to this article can be found online at https://doi.org/10.1016/j.socscimed.2025.118335.

Supplementary File 1

Supplementary File 2

Supplementary File 3

## Figures and Tables

**Table 1:** Participant characteristics.

	YOUNG PEOPLE	PARENT/GUARDIAN	BEREAVED FAMILY MEMBERS	PARTICIPANTS IN TOTAL
	PARTICIPANTS (n=6, 29 %^a^)	PARTICIPANTS (n=6, 29 %)	PARTICIPANTS (n=9, 43 %)	(n=21, 100 %)
***YOUNG PERSON AGE GROUP (YEARS)***				
*14*–*17*	3 (14 %)	1 (5 %)	2 (10 %)	6 (29 %)
*18*–*25*	3 (14 %)	5 (24 %)	7 (33 %)	15 (71 %)
***YOUNG PERSON SEX***				
*Male*	3 (14 %)	3 (14 %)	3 (14 %)	9 (43 %)
*Female*	3 (14 %)	3 (14 %)	6 (29 %)	12 (57 %)
***YOUNG PERSON ETHNICITY***				
*White British*	4 (19 %)	4 (19 %)	6 (29 %)	14 (67 %)
*Black*	–	–	3 (14 %)	3 (14 %)
*Asian*	1 (5 %)	2 (10 %)	–	3 (14 %)
*Other*	1 (5 %)	–	–	1 (5 %)
***YOUNG PERSON DEPRIVATION LEVEL (Indices of Multiple Deprivation)***				
*1*-*3 (most deprived)*	3 (14 %)	2 (10 %)	6 (29 %)	11 (52 %)
4–7	1 (5 %)	4 (19 %)	1 (5 %)	6 (29 %)
*8*-*10 (least deprived)*	2 (10 %)	–	2 (10 %)	4 (19 %)
***YOUNG PERSON HEALTH CONDITION***				
*Congenital*	2 (10 %)	3 (14 %)	1 (5 %)	6 (29 %)
*Metabolic*	1 (5 %)	1 (5 %)	–	2 (10 %)
*Cancer*	1 (5 %)	–	5 (24 %)	6 (29 %)
*Circulatory*	–	–	1 (5 %)	1 (5 %)
*Neurological*	1 (5 %)	1 (5 %)	2 (10 %)	4 (19 %)
*Respiratory*	1 (5 %)	1 (5 %)	–	2 (10 %)
***RECRUIMENT METHOD***				
*Charities*	1 (5 %)	1 (5 %)	3 (14 %)	5 (24 %)
*Hospices*	2 (10 %)	4 (19 %)	–	6 (29 %)
*Social media (Twitter and Facebook)*	2 (10 %)	1 (5 %)	4 (19 %)	7 (33 %)
*Snowball*	1 (5 %)	–	2 (10 %)	3 (14 %)

a (Approximate percentage values leading to rounding errors above).

## Data Availability

The data that has been used is confidential.
