# Supplementary material for: Understanding what matters to young people with life-limiting and life-threatening conditions: a qualitative study to inform the development of a young person end-of-life capability measure
Source: Soc Sci Med. Author manuscript; Available in PMC 2026 Feb 20. (PMC7618747; doi:10.1016/j.socscimed.2025.118335)
Supplement: Supplementary File 1 [file EMS212370-supplement-Supplementary_File_1.docx]

**Supplementary File 1: COREQ checklist for study reporting**

| **Domain** | **Item number** | **Comment** | **Reported on page number or not applicable (N/A)** |
| --- | --- | --- | --- |
| **Domain 1: Research team and reflexivity** |  |  |  |
| *Personal characteristics* |  |  |  |
| Interviewer/facilitator | 1 | [interviewer initials] conducted the interviews | 9 |
| Credentials | 2 | [interviewer initials and credentials] | 9 |
| Occupation | 3 | [interviewer initials and occupation details] | 9 |
| Gender | 4 | [interviewer initials and gender details] | 9 |
| Experience and training | 5 | [interviewer initials] had previous experience working and undertaking research with vulnerable groups and received training on conducting interviewing on sensitive topics. | 9 |
| *Relationship with participants* |  |  |  |
| Relationship established | 6 | The interviewer and broader research team had no relationships with participants prior to the commencement of the study and recruitment. | N/A |
| Participant knowledge of the interviewer | 7 | Interviewer introduced themselves at the beginning of the interviews, explained their role, occupation, and purpose of the study. The interviewer answered any questions participants may have had about the study and those involved in it. | N/A |
| Interviewer characteristics | 8 | As above – see item numbers 2, 3, 4, 5 | 9 |
| **Domain 2: Study design** |  |  |  |
| *Theoretical framework* |  |  |  |
| Methodological orientation and theory | 9 | Data collection and analysis were undertaken concurrently using constant comparison. | 6 |
| *Participant selection* |  |  |  |
| Sampling | 10 | A combination of maximum variation approach to sampling, convenience sampling (due to practical limitations) and snowball sampling was used. | 7-8 |
| Method of approach | 11 | Multiple recruitment avenues were used to recruit participants across the UK. Online and printed adverts were used, as well as a study newsletter circulated via staff members, by email and/or in-person, in child (four) and adult (one) hospices, charitable organisations (six) and through social media using Twitter posts, relevant support groups on Facebook and the Facebook ‘targeted ads feature’. | 8 |
| Sample size | 12 | Twenty-one | 2, 12 |
| Non-participation | 13 | Forty-five people expressed initial interest in participating, from which twenty-one interviews were undertaken. The most common reasons for individuals not being included in the study after expressing initial interest were that they did not fit the age criteria (n=6), their condition was not a LLC/LTC (n=7) and the bereavement period was less than six months (n=2). Some did not respond to follow-up contact (n=8). | 12 |
| *Setting* |  |  |  |
| Setting of data collection | 14 | Most interviews were undertaken online and video and audio-recorded. Some participants, however, preferred a telephone interview and this was also facilitated. At the time of their interview, participants were located in either home or care settings. | 9 |
| Presence of nonparticipants | 15 | All young people were given the option of parents being present, for example to assist with using technology to take part in the online interview and to interpret non-verbal communication. Parents were present in two interviews with young people. | 9, 13 |
| Description of sample | 16 | Interviews were undertaken with six young people, six parents and nine bereaved family members (parents and siblings). Participant characteristics are fully presented in Table 1. Interviews captured the experiences of young people from diverse backgrounds, including those from the most deprived areas (n=11), with a wide range of conditions, illness trajectories and types of care received. | 12-13, 36 |
| *Data collection* |  |  |  |
| Interview guide | 17 | Three separate topic guides were used (Supplementary file 2). The topic guide for young people participants asked questions about their health condition and aspects of life that were important to them and why. The topic guides for family member participants asked questions about the young person’s condition and what aspects of life they thought were important to the young person and why. | 9 |
| Repeat interviews | 18 | No repeat interviews were undertaken | N/A |
| Audio/visual recording | 19 | Most interviews were undertaken online and video and audio-recorded. Some participants, however, preferred a telephone interview and this was also facilitated and audio-recorded only. | 9 |
| Field notes | 20 | Reflections were recorded after each interview and discussed at regular debrief meetings with members of the research team. | 12 |
| Duration | 21 | Interviews with young people lasted between 27 and 47 (mean 37) minutes; those with family members lasted between 11 and 68 (mean 44) minutes. | 13 |
| Data saturation | 22 | Recruitment ended when the conceptual attributes were deemed to be fully developed (J. Coast et al., 2012; Owen-Smith & Coast, 2017). | 8 |
| Transcripts returned | 23 | Transcripts were not returned to participants and were only seen by the research team. | 10 |
| **Domain 3: analysis and findings** |  |  |  |
| *Data analysis* |  |  |  |
| Number of data coders | 24 | Three – [initials of coders]  Analysis was undertaken primarily by [researcher initials] with support from research team members [initials of team members]. Early transcripts were coded by [initials of coders] | 10 |
| Description of the coding tree | 25 | Early transcripts were coded on a line-by-line basis (open coding) to identify emerging themes. Codes were developed in a hierarchical manner, comparing data for similarities and differences (axial coding). Three separate coding schedules alongside spider diagrams were developed for each group of participants to map themes and sub-themes included in the schedules. | 10 |
| Derivation of themes | 26 | Themes derived from the data – inductive approach. | 10 |
| Software | 27 | NVivo12 qualitative data analysis software was used to analyse the data | 10 |
| Participant checking | 28 | No | N/A |
| *Reporting* |  |  |  |
| Quotations presented | 29 | Yes – all quotations were identified by participant group and number. All findings were illustrated with relevant quotations. | 14-25 |
| Data and findings consistent | 30 | Yes – all data was interpreted in the Discussion section in relation to existing literature and novel findings. | 25-28 |
| Clarity of major themes | 31 | Yes – major themes were clearly identified in the findings section. | 14-25 |
| Clarity of minor themes | 32 | Yes – minor themes were discussed as part of each major theme. Minor instances of differing perspectives across participant groups were identified and reported as part of the relevant themes. | 14-25 |
