# Supplementary material for: Understanding what matters to young people with life-limiting and life-threatening conditions: a qualitative study to inform the development of a young person end-of-life capability measure
Source: Soc Sci Med. Author manuscript; Available in PMC 2026 Feb 20. (PMC7618747; doi:10.1016/j.socscimed.2025.118335)
Supplement: Supplementary File 2 [file EMS212370-supplement-Supplementary_File_2.docx]

**Interview topic guide for young people**

***Introduction:***

**• Could you tell me a bit about yourself? (Hobbies/interests/Likes and Dislikes)**

***Questions on young person’s condition:***

• **Could you tell me a bit about the condition you have?**

o When did you find out about it/how?

o How does the condition affect your day-to-day life?

o What support have you been receiving?

***Questions about things that are important in the young person’s life:***

**• Could you tell me about what is important to you in life right now…..**

**• You talked about** …

o How important is that to you? / Why?

o What else is important to you in your life right now? Why?

**• Do you think that what is important to you has changed since….? /How?/ Do you think that what is important to you may change going forward?**

**• Do you think that the things that are important to you have changed since the pandemic started? How?**

**• What would you find helpful right now? How would it help?**

**Interview topic guide for parent/guardian**

***Introduction***

**• Could you tell me a bit about yourself/your child? (Hobbies/Interests)**

***Questions on young person’s condition:***

**• Could you tell me a bit about the condition your child has?**

o When did you/they find out about it and how?

o How does the condition affect their day-to-day life?

o What support have they been receiving?

***Questions about things that are important in the young person’s life:***

**• Could you tell me about what you feel is important to your child right now…**

**• You talked about…**

o How important is that to your child? / Why?

o What else is important in their life right now? Why?

**• Do you think that what is important to your child has changed since….? /How?/ Do you think that what is important to your child may change going forward?**

**• Do you think that the things that are important to your child have changed since the pandemic started? How?**

**• What would they find helpful right now? How would it help?**

**Interview topic guide for bereaved family members**

***Introduction***

**• Could you tell me a bit about yourself?**

**• Could you tell me a bit about the young person you were close to?**

o Relationship to young person; age of young person; time spent with them, and type of support/care provided to young person

***Questions on young person’s condition:***

**• Could you tell me a bit about the condition the young person had?**

o When did you/they find out about it and how?

o How did the condition affect their day-to-day life?

o What support and care did they receive?

***Questions about things that are important in the young person’s life:***

**• Could you tell me about what you feel was important to the young person near the end of their life?**

**• You talked about…**

o How important was that to the young person / Why?

o What else was important in their life? Why?

**• Do you think that what was important to the young person changed over time? /How?**
