# Supplementary material for: Understanding what matters to young people with life-limiting and life-threatening conditions: a qualitative study to inform the development of a young person end-of-life capability measure
Source: Soc Sci Med. Author manuscript; Available in PMC 2026 Feb 20. (PMC7618747; doi:10.1016/j.socscimed.2025.118335)
Supplement: Supplementary File 3 [file EMS212370-supplement-Supplementary_File_3.docx]

Table 1 - Dimensions of CYP measures

| PedsQL^1^ | EQ-5D-Y^2^ | CHU-9D^2^ | ICECAP-CYP: Age 11-15^3^ | C-POS^4^ |
| --- | --- | --- | --- | --- |
| - Physical - Emotional - Social - School functioning | - Mobility - Self-care - Usual activities - Pain or discomfort - Anxiety or depression | - Worried - Sad - Pain - Tired - Annoyed - Schoolwork - Sleep - Daily routine - Activities | - Fun and enjoyment - Learning and experiencing - Attachment - Emotional security - Achievement - Identity and choice - Physical safety - Aspiration | - Physical - Emotional - Psychological - Spiritual - Information and support |

^1^ PedsQL (Varni et al., 1999): HRQoL CYP measure commonly used in PPC

^2^ EQ-5D-Y (Wille et al., 2010) and CHU-9D (Stevens, 2010): HRQoL CYP measures commonly used with CYP

^3^ ICECAP-CYP: Age 11-15 (Husbands et al., 2024): CYP capability measure for use in economic evaluation

^4^ C-POS (Coombes et al., 2024): HRQoL measure for CYP with LLCs/LTCs

Table 2 - Dimensions of adult PC and EoL measures

| POS-E ^1^ | ICECAP-SCM ^2^ |
| --- | --- |
| - Family anxiety - Pain - Other symptoms - Depression - Anxiety - Practical matters - Feeling good | - Choice - Love and affection - Physical suffering - Emotional suffering - Dignity - Being supported - Preparation |

^1^ POS-E (Dzingina et al., 2017): an adult PC measure developed for use in economic evaluation

^2^ ICECAP-SCM (Sutton & Coast, 2014): an adult EoL capability measure

**References:**

Coombes, L., Braybrook, D., Harðardóttir, D., Scott, H.M., Bristowe, K., Ellis-Smith, C., et al. (2024). Cognitive testing of the Children’s Palliative Outcome Scale (C-POS) with children, young people and their parents/carers. *Palliative Medicine*, 02692163241248735.

Dzingina, M., Higginson, I.J., McCrone, P., & Murtagh, F.E.M. (2017). Development of a Patient-Reported Palliative Care-Specific Health Classification System: The POS-E. *The patient,* 10, 353-365.

Husbands, S., Mitchell, P.M., Kinghorn, P., Byford, S., Breheny, K., Bailey, C., et al. (2024). The development of a capability wellbeing measure in economic evaluation for children and young people aged 11-15. *Social Science & Medicine,* 360, 117311.

Stevens, K.J. (2010). Working With Children to Develop Dimensions for a Preference-Based, Generic, Pediatric, Health-Related Quality-of-Life Measure. *Qualitative Health Research,* 20, 340-351.

Sutton, E.J., & Coast, J. (2014). Development of a supportive care measure for economic evaluation of end-of-life care using qualitative methods. *Palliative Medicine,* 28, 151-157.

Varni, J.W., Seid, M., & Rode, C.A. (1999). The PedsQL™: Measurement Model for the Pediatric Quality of Life Inventory. *Medical Care,* 37, 126-139.

Wille, N., Badia, X., Bonsel, G., Burström, K., Cavrini, G., Devlin, N., et al. (2010). Development of the EQ-5D-Y: a child-friendly version of the EQ-5D. *Qual Life Res,* 19, 875-886.
